# Expression of Network Medicine-Predicted Genes in Human Macrophages Infected with *Leishmania major*

**DOI:** 10.3390/ijms252212084

**Published:** 2024-11-11

**Authors:** Felipe Caixeta, Vinicius Dantas Martins, Amanda Braga Figueiredo, Luis Carlos Crocco Afonso, Paolo Tieri, Filippo Castiglione, Leandro Martins de Freitas, Tatiani Uceli Maioli

**Affiliations:** 1 Programa Interunidades de Pós-Graduação em Bioinformática, Universidade Federal de Minas Gerais, Belo Horizonte 31270-901, MG, Brazil; caixeta.felipe@gmail.com; 2 Cedars Sinai, Biomedical Sciences, Los Angeles, CA 90048, USA; vdantasmartins@gmail.com; 3 Programa de Pós-Graduação em Bioquímica e Imunologia, Universidade Federal de Minas Gerais, Belo Horizonte 31270-901, MG, Brazil; 4 Instituto Israelita de Ensino e Pesquisa, Hospital Israelita Albert Einstein, São Paulo 05652-000, SP, Brazil; amanda.figueiredo@accamargo.org.br; 5 Departamento de Biologia, Instituto de Ciências Biológicas e Exatas, Universidade Federal de Ouro Preto, Ouro Preto 35402-136, MG, Brazil; 6 CNR—Consiglio Nazionale delle Ricerche, IAC Istituto per le Applicazioni del Calcolo, 00185 Rome, Italy; paolo.tieri@cnr.it (P.T.); filippo.castiglione@gmail.com (F.C.); 7 Núcleo de Biointegração, Instituto Multidisciplinar em Saúde, Universidade Federal da Bahia, Vitóriada Conquista 45029-094, BA, Brazil; leandromartins@ufba.br; 8 Departamento de Nutrição, Universidade Federal de Minas Gerais, Belo Horizonte 31270-901, MG, Brazil

**Keywords:** *Leishmania*, gene expression, macrophages, predicted genes

## Abstract

*Leishmania* spp. commonly infects phagocytic cells of the immune system, particularly macrophages, employing various immune evasion strategies that enable their survival by altering the intracellular environment. In mammals, these parasites establish persistent infections by modulating gene expression in macrophages, thus interfering with immune signaling and response pathways, ultimately creating a favorable environment for the parasite’s survival and reproduction. In this study, our objective was to use data mining and subsequent filtering techniques to identify the genes that play a crucial role in the infection process of *Leishmania* spp. We aimed to pinpoint genes that have the potential to influence the progression of *Leishmania* infection. To achieve this, we exploited prior, curated knowledge from major databases and constructed 16 datasets of human molecular information consisting of coding genes and corresponding proteins. We obtained over 400 proteins, identifying approximately 200 genes. The proteins coded by these genes were subsequently used to build a network of protein–protein interactions, which enabled the identification of key players; we named this set Predicted Genes. Then, we selected approximately 10% of Predicted Genes for biological validation. THP-1 cells, a line of human macrophages, were infected with *Leishmania major* in vitro for the validation process. We observed that *L. major* has the capacity to impact crucial genes involved in the immune response, resulting in macrophage inactivation and creating a conducive environment for the survival of *Leishmania* parasites.

## 1. Introduction

Leishmaniasis is a spectrum of vector-borne parasitic diseases caused by unicellular protozoa of the genus *Leishmania*. They remain a significant public health burden, with an estimated 12 million infected individuals worldwide and approximately 350 million people at risk [1]. Despite extensive research efforts and accumulated knowledge, Leishmaniasis continues to be classified as an emerging or re-emerging disease with complex control challenges. In response to the ongoing challenges and global needs, scientific research on Leishmaniasis has focused on acquiring new knowledge and developing effective control measures [2]. The intricate immune response to *Leishmania* infection remains incompletely understood, but the parasite’s remarkable ability to outsmart host immune cells is a key factor in its pathogenicity [3]. *Leishmania*’s strategies extend beyond mere survival within macrophages. Their interaction triggers the release of antigenic particles, stimulating the host’s adaptive immune response [4]. Macrophages, however, stand as the parasite’s preferred sanctuary while playing a central role in shaping the immune response, highlighting their critical role in Leishmaniasis pathogenesis. These phagocytic cells serve as the parasite’s primary target and battleground. Notably, *L. major* is frequently utilized in experimental models, providing well-characterized in vivo and in vitro insights into disease development [5]. Deciphering this complex interplay, including the role of different macrophage phenotypes and their interactions with *Leishmania*, holds immense promise for developing novel therapeutic strategies against this persistent parasitic disease. *Leishmania* infection, along with the parasite’s capacity to evade the host’s immune response, involves alterations in gene expression within both the host cells and the parasite itself. Within a genome, well-defined gene expression control mechanisms regulate those genes that must be expressed by the cell at a given time in response to internal or external factors [6,7,8]. Gene expression profiling has been used in several studies on pathogenic microorganisms, including parasitic protozoa such as *Leishmania* [6,9]. This technique has allowed researchers to identify differentially expressed genes at distinct stages of infection, leading to a better understanding of the sequence of gene activation and its association with critical events at each stage. Changes in the expression of genes encoding STAT2, IL-18, and CXCL2, as well as genes important in the steroid and cholesterol biosynthesis pathway, have already been identified in infected cells [9]. As in mice, the gene expression of human macrophages can be differentiated into susceptibility or resistance profiles when they become infected with *L. major* [10]. In this regard, the study of the interactome, i.e., the whole set of protein–protein interactions (PPIs) in a specific cell or organism, would be a valuable resource for understanding the molecular basis of Leishmaniasis, identifying new targets for developing drugs and designing synthetic biology circuits. However, assembling the interactome is a daunting task, as it requires identifying and characterizing all protein–protein interactions in a given system [11]. Recent advances in high-throughput technologies have uncovered interesting aspects of *Leishmania* infection, and details of the underlying protein–protein interactions have brought us closer to the goal of assembling the interactome, a comprehensive map of all protein–protein interactions in a cell or organism [12,13]. However, there are still many challenges to overcome, such as the need to validate protein–protein interactions, the abundance of false negatives, and to understand the functional significance of these interactions. Despite these challenges, the interactome is a goal of inestimable value with the potential to revolutionize our understanding of biology and medicine. These methodologies have converged in the search for hypotheses that can be tested in silico, in vitro, and finally in vivo in clinical studies that can support the research data [14]. In view of the increasing availability of data on Leishmaniasis, the present work was focused on performing data mining of public databases that provide a wide spectrum of different results and methodologies. The goal was to search for results on Leishmaniasis in general, l eading to filtering and treatment of the data to assemble a protein–protein interaction network with the DIAmond algorithm to identify gene modules and consequent analysis of the centrality and importance of the genes in the network. To confirm these in silico-generated data, some genes were then selected for biological validation. The parasite–host interaction of Leishmaniasis has been studied by many models, including computational models that describe the dynamics of the parasite and macrophages in the early phase of the immune response [15]. Recent findings in the field of data mining have also shown the power of this approach [16]. However, no work has yet been performed on database integration mining for *Leishmania*sis, as in this study, in which we looked for information about Leishmaniasis in several databases.

## 2. Results

### 2.1. Data Sets, Gene Identification and Enrichment

We utilized the following databases: GWAS, UniProt, Malacards, DisGeNET, HumanMine, NCBI, and GeO from cells infected with *Leishmania*, alongside a search for the most cited proteins produced upon *Leishmania* infection relevant articles. This approach allowed us to amass a large number of proteins and genes associated with Leishmaniasis.

We conducted a manual search of the known literature. In all databases, searches were made using the keyword “Leishmaniasis”. We then obtained proteins and genes, which were organized only with human datasets (HDs) according to the origin of the data, the reference, and the proteins found in the results (Table S1). The search and assembly of the HDs generated 439 proteins, which had identification codes (IDs). After checking the protein coded by each gene, gene symbols were identified and confirmed using UniProt and HGNC databases. The duplicate genes, proteins that were not associated with a gene symbol, and proteins from different databases/studies were filtered out. After filtering, we were left with 287 genes associated with the 439 proteins.

**Figure 1:**
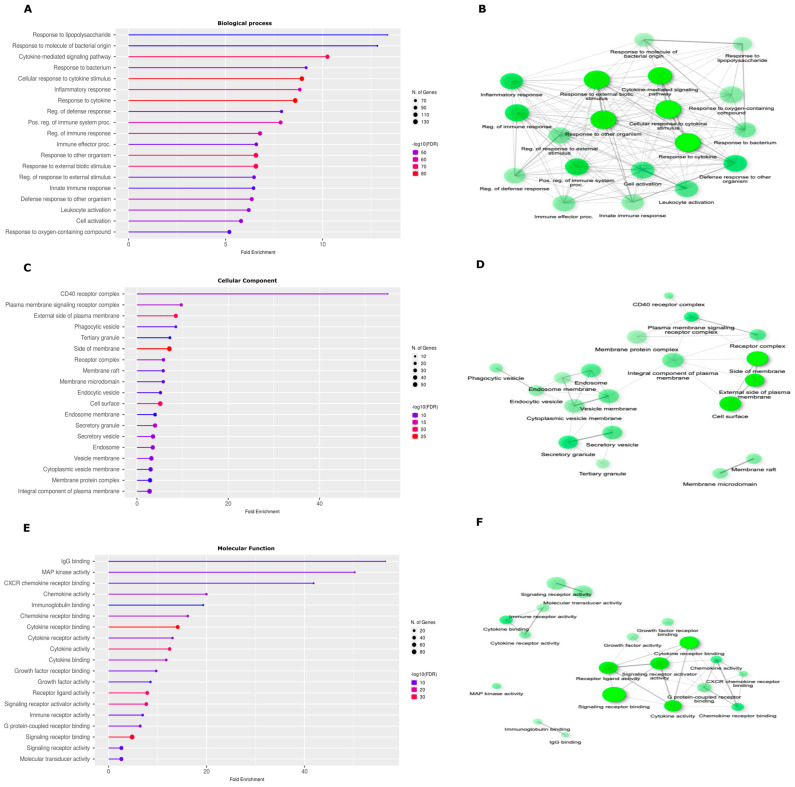
Enrichment analysis of 287 source genes. (**A**) Biological process. (**B**) Network relationship between enriched BP. (**C**) Cellular component. (**D**) Network relationship between enriched CC. (**E**) Molecular function. (**F**) Network relationship between enriched MF. Enrichment analysis was performed using ShinyGO with a false discovery rate (FDR) cutoff of 0.05. Top pathways were then identified based on FDR and further ranked by fold enrichment. Highly similar pathways sharing over 95% gene overlap were consolidated, with the most statistically significant one representing the group. The plotted network visualizes the interconnectedness of the enriched pathways. Edges connect GO terms (nodes) if they share at least 20% of their genes, with darker nodes marking greater enrichment and the size of the nodes representing the number of genes. Thicker edges indicate a higher degree of gene overlap between connected pathways.

The identification procedures and filtering resulted in a high-quality dataset of genes and proteins related to leishmaniasis. After identifying 287 genes (Table S2), they were used in the Cytoscape system for the assembly of PPI networks and gene ontology enrichment analysis. The gene set enrichment analysis returned pathways related to immune response. To go deeper into the biological significance and functional relationships of the 287 genes identified in our initial analysis, we performed an enrichment analysis. This revealed an intriguing association of the identified genes with specific biological process 1 (Figure 1) and with Leishmaniasis (Figure S1). Notably, significant enrichment was observed in pathways related to cytokine signaling. Furthermore, the gene set exhibited enrichment in terms related to broader biotic interactions, such as “response to other organisms” and “response to external biotic stimuli” (Figure 1A). These findings suggest a potential role for these genes in processes involving inflammatory signaling and inter-organismal interactions. To further illuminate the overarching themes within the enriched pathways, we employed a network-based analysis of Gene Ontology (GO) terms, and this facilitates the identification of overarching themes that are not readily apparent from individual pathway enrichments (Figure 1B,D,F). The examination of cellular components reveals the crucial role of the external plasma membranes, the cell surface itself, and their combined interaction in constituting key signaling pathways within source genes (Figure 1C,D). Scrutiny of molecular functions within our source genes reveals a striking enrichment for terms associated with “cytokine receptor interactions”, “activity”, and “signaling pathways”. This pattern suggests the emergence of cytokine signaling as a critical overarching functional module within these genes (Figure 1E,F). These findings underscore the pivotal role of cytokine signaling in shaping the overall functional landscape of genes expressed by *Leishmania*-infected cells.

### 2.2. Assembly of Interactome for Predicted Genes Identification with DIAmonD Algorithm

**Figure 2:**
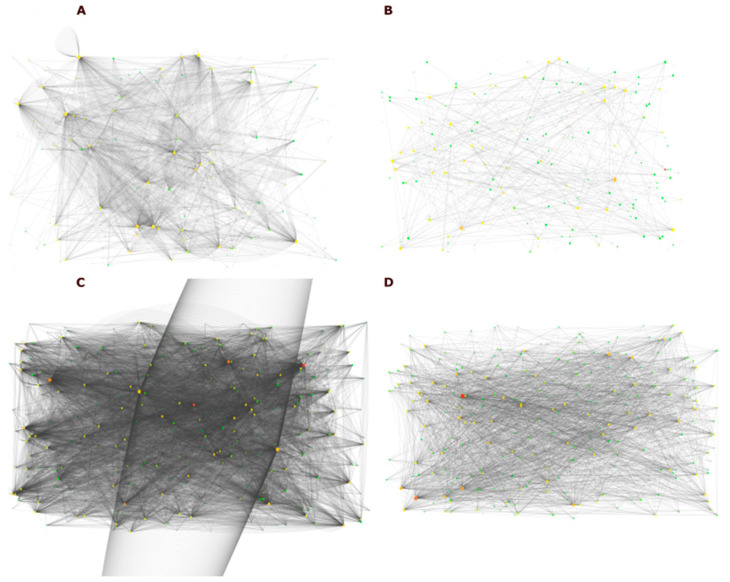
Human interactomes in response to Leishmaniasis. (**A**) BioGrid interactome was assembled by importing source genes from PPI data from the BioGrid database, resulting in an interactome with 259 nodes and 1727 edges, demonstrating the high linkage and high number of interactions between the source genes. (**B**) APID interactome was assembled by importing source gene data from various sources, including PPI databases, expression datasets, and GO annotations. This interactome contains 253 nodes and 418 edges, showing the interactions between the proteins encoded by the source genes. (**C**) DIAmond interactome to the genes derived from the BioGrid plug-in resulted in an increased number of genes and edges within the network, resulting in 300 nodes and 11,741 edges. (**D**) DIAmond APID-derived interactome also yielded an augmented number of nodes and edges, identifying 300 nodes and 2929 edges. Red nodes are the most connected, followed by yellow and green.

To identify the relationship between the 287 genes related to the 439 proteins under scrutiny, we used Cytoscape [17] to build the PPI network using the plug-ins APID2NET [18] and BioGrid [19]. We then obtained two complex interactomics corresponding to the module of our interest originating from two different plug-ins, BioGrid and APID [20]. The BioGrid interactome was assembled by importing PPI data from the BioGrid database. This interactome contains 259 nodes and 1727 edges, demonstrating the high linkage and high number of interactions between the source genes (Figure 2A). The APID interactome was assembled by importing data from various sources, including PPI databases, expression datasets, and GO annotations. This interactome contains 253 nodes and 418 edges, showing the interactions between the proteins encoded by the source genes (Figure 2B). The two interactomes we assembled provide complementary views of the PPI network between the source genes. The BioGrid interactome is more comprehensive regarding the number of PPIs, while the APID interactome includes additional information, such as expression data and GO annotations. Combining these two interactomes provided a more complete view of the PPI network and its relationship to other molecular states.

### 2.3. Identification of Predicted Genes by DIAmonD Algorithm

To investigate the connectivity patterns of protein interactions, we leveraged the well-established connection of the plug-ins with databases of protein interactions. We have opted to employ the predictive algorithm DIAmond [21]. This algorithm facilitates the assessment of the significance of the connectivity between immediate neighbors of proteins by iteratively expanding the module; it then adds the most significantly connected node to the module, increasing the module by one node at a time. We used DIAmond to identify special modules of genes that are involved in Leishmaniasis, namely, modules that are highly interconnected in the two assembled interatomics (APID and BioGridinteractomes). The output of DIAmond was selected to be 300 genes, which is compatible with the literature stated by Ghiassian [21]. This number is reasonable, considering that our original dataset of interest contained 287 genes. However, the two results did share some common genes. Applying DIAmond to the genes derived from the BioGrid plug-in resulted in an increased number of genes and edges within the network, resulting in 300 nodes and 11,741 edges (Figure 2C). This led to the formation of a denser interactome.

Similarly, applying DIAmond to the APID-derived interactome also yielded an augmented number of nodes and edges, identifying 300 nodes and 2929 edges (Figure 2D). When we overlapped the results of the 300 genes obtained from both interactomes, we identified 217 genes with higher centrality in both the APID and BioGrid interactomes. We call them “Predicted Genes”. The two plug-ins are different from each other, but not so much, which makes the result of 217 genes (Table S2) compatible with what was expected. Further study of the Predicted Genes is needed to understand their role in Leishmaniasis.

### 2.4. The Predicted Genes Are Expressed by Human Macrophages Infected with L. major

After identifying the Predicted Genes from both the APID and BioGrid plug-ins, we compared the categorizations generated by the results of the two plug-ins. APID and BioGrid generated ranked results based on the centrality values of these genes in the networks, generating a ranking of the 217 genes. The comparison of the results from the two plug-ins allowed us to select the genes of interest more precisely. The shared genes found in the DIAmond module suggest that Predicted Genes are strong candidates for the category of central genes regulating the response to leishmaniasis. To establish the significance of these genes in controlling leishmaniasis, with macrophages as the reference cell, we needed to further validate the Predicted Genes found by the DIAmonD algorithm as core genes.

We selected the 22 most prominent genes for this validation (see Table S3).

**Figure 3:**
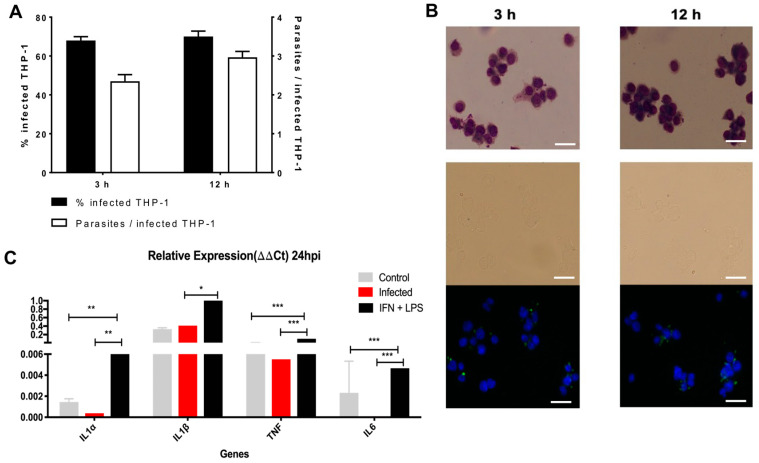
THP-1 infected with *L. major* do not show increased concentrations of inflammatory cytokines genes. (**A**) Quantification of THP-1 cells infected with *L. major* in 3 h post-infection and 12 h post-infection. (**B**) Corresponding to the amastigote forms already inside the parasitophorous vacuole formed in the macrophages in 3 h post-infection and 12 h post-infection, the green dots are *L. major* amastigotes labeled with CFSE, and blue are macrophages labeled with DAPI. Scale bar = 20 µm. (**C**) Relative expression of some inflammatory cytokines in control, infected, and IFN-gamma and LPS-stimulated THP-1 cells for 24 h. Significant differences were determined by using Student’s *t*-test (* *p* < 0.05, ** *p* < 0.01, *** *p* < 0.001).

To ensure consistency with our gene selection, we employed an in vitro model of human macrophage infection using THP-1 cells [22] infected by *L. major*, a *Leishmania* species most used in experimental infection [23,24]. THP-1 cells were effectively infected (Figure 3A,B) with the metacyclic parasites of *L. major*, thus demonstrating that THP-1-derived macrophages are functional hosts for the parasite [25]. To confirm the biological validation data, we first analyzed the expression of four cytokine genes related to the innate immune response, well known to be produced by resistant macrophages to *L. major* (IL-1alfa, IL-1beta, TNF-alfa, and IL-6). INF-gamma and LPS were used as a positive control for activated macrophages. As expected, the results showed that THP-1 cells stimulated with IFN-gamma and LPS for 24 h had higher expression of inflammatory cytokines compared to non-infected cells. However, the THP-1 infected with *L. major* for 24 h had no increase in the expression of those cytokines, demonstrating THP-1 cells as a model for susceptibility to *L. major* infection (Figure 3C).

**Figure 4:**
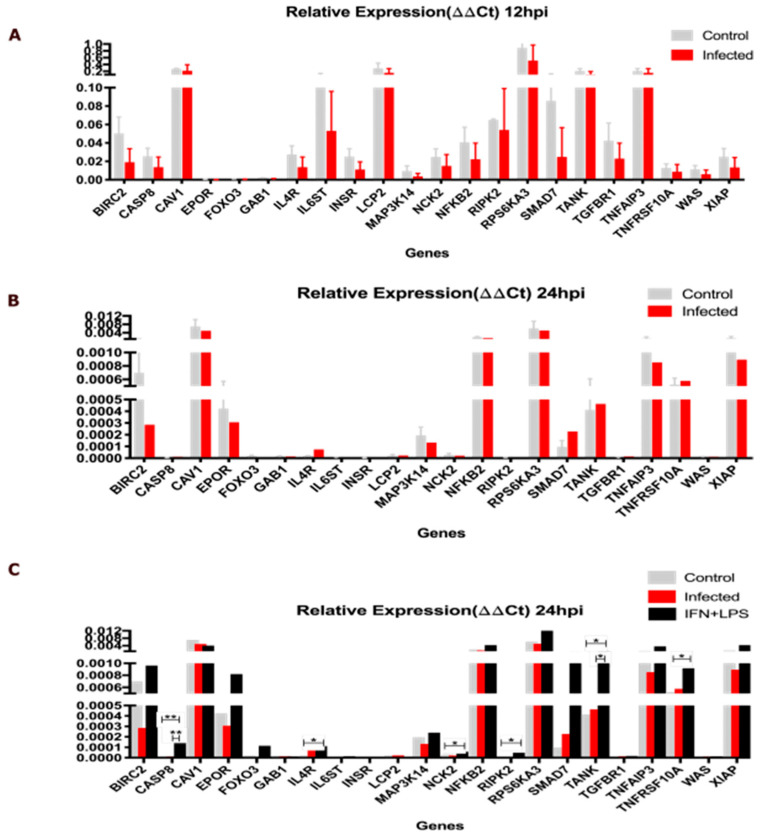
Relative expression of Predicted Genes in THP-1 cells infected with *L. major*. (**A**) Relative expression of 22 most prominent Predicted Genes at 12 h post-infection (hpi) with *L major* in control and infected THP-1 cells. (**B**) Relative expression of 22 most prominent Predicted Genes at 24 hpi with *L. major* in control and infected THP1 cells. (**C**) Analysis of gene expression at 24 hpi following combined interferon and LPS stimulation confirmed the response of THP-1 cells to the stimulus; statistically significant differences were observed in a subset, including CASP8, IL4R, NCK2, Ripk2, TANK, and TNFRSF10A. Notably, these differences were present only when comparing the combined stimulus group to both the unstimulated control and the infected groups. Specifically, CASP8, IL4R, NCK2, TANK, and TNFRSF10A showed differences compared to the control, while CASP8 and TANK displayed additional unique differences compared to the infected group. Significant differences were determined by using Student’s *t*-test (* *p* < 0.05, ** *p* < 0.01).

We opted to perform RT-qPCR experiments at 12 and 24 h post-infection. The results obtained at 12 h post-*L. major* infection show the expression of several of the Predicted Genes during the early stage of the infection. However, none of these genes exhibited significant differential expression compared to non-infected cells (either over- or under-expressed) at the assessed time point 4 (Figure 4A). We then investigated the expression of the Predicted Genes in THP-1 cells infected with *L. major* at 24 h post-infection and found that these genes were still expressed but at lower levels than those found after 12 h of infection. No significant differences in the expression of the analyzed genes (neither over- nor under-expressed) were found between control and infected cells at this time point (Figure 4B). Upon analyzing the expression of the same 22 genes at 24 hpi under IFN-gamma plus LPS stimulation (Figure 4C), we confirmed the reactivity of THP-1 cells to the stimulus. We found statistically significant differences in expression for some genes (specifically, CASP8, IL4R, NCK2, Ripk2, TANK, and TNFRSF10A) compared to non-stimulated cells and to *L. major* infected cells. Again, we observed that *L. major* can induce decreased gene expression in THP-1 cells, showing a profile compatible with susceptibility to *Leishmania* infection. In short, the identified Predicted Genes can be a good target to modulate the immune response to *Leishmania* infection.

## 3. Discussion

*Leishmania* is an obligate intracellular parasite that can subvert the host immune response in its favor affecting macrophage response mechanisms such as apoptosis, oxidative damage, subversion of the immune response, and modification of nutrient availability [26,27,28]. To evaluate the genes involved in the parasite–host relationship, new methodologies are needed. In the present study, we searched databases for genes related to leishmaniasis and then filtered the results only for those human genes associated with macrophages. We thus turned to understand the parasite–host response from the point of view of gene expression by macrophages in an attempt to build an omics model of the parasite–host relationship. Similar relatively recent studies have shown satisfactory results in the literature on data mining [29]. Due to the pivotal role of protein–protein interactions (PPIs) in the pathogenesis of diseases, the manipulation of protein–protein complexes holds significant clinical relevance. The effective design of compounds influencing PPIs necessitates a comprehensive understanding of the molecular intricacies of the involved protein–protein system. Although there has been progress in data mining and in the use of interactomes as study tools, the biological validation of these data remains crucial. When we started this step, it was expected that many Predicted Genes analyzed would be differentially expressed by infected cells when compared to uninfected cells. Surprisingly, the genes analyzed at both infection times showed the same behavior in infected and uninfected cells. This fact could be linked with the susceptibility to *Leishmania*. The successful infection and ultimate dissemination of parasites throughout the body may depend on the early interactions between the parasite and the host cell [30]. Genotypic differences between the same *Leishmania* species can lead to detectable differences in macrophage responses [31]. While the expression of certain genes in macrophages in response to pathogens has been described, our findings for CASP8 present an intriguing contrast. Previous studies have reported reduced CASP8 expression in THP-1 cells infected with *L. infantum* [32], suggesting a potential role in apoptosis inhibition. Also, there is an increase in CASP8 mRNA expression in THP-1 cells infected with *L. tarentolae* [33]. What differs from our data is that CASP8 mRNA increases only in cells stimulated with IFN-gamma and LPS.

This discrepancy highlights the complexity of the relationship between *Leishmania* infection and CASP8 expression, which may be influenced by factors such as the susceptibility of the cells related to different pathogen species. Consistent with previous findings by Moore and Matlashewski [34] and Valdés-Reyes et al. [35], *Leishmania* may employ strategies to inhibit apoptosis in macrophages and dendritic cells, potentially explaining the reduced CASP8 levels observed in THP-1 cells. This manipulation of host cell signaling could allow the parasite to evade the immune response. IL4R, an interleukin-4 receptor, is another gene of interest that appears to be downregulated in persistent *Leishmania* infections [36]. Additionally, NCK2, TANK, and TNFRSF10A are noteworthy genes with limited existing knowledge regarding their involvement in Leishmania infection. Further exploration of these genes could provide valuable insights into the host–parasite interaction. *Leishmania* infection has already been well described and characterized in THP-1 cells. This model has been used for more than two decades [23,37]. THP-1 cells differentiate into human macrophages and are, therefore, a good model for the study of parasites that infect this cell type, such as *Leishmania*. These cells have also been used in studies on leishmanicidal drugs and inhibitors against *Leishmania* [37,38]. Another use of these cells was in studies that demonstrated modification in gene expression of proteins related to gene transcription, RNA editing, histones, DNA repair and replication, and also proteins involved in cell survival and signal transduction [39] in addition to proteomic and other omics studies even demonstrating their altered response to infection with different pathogens [22]. All these successful applications demonstrate the acceptance and practicality that this model offers. Therefore, susceptible and resistant macrophages show a different expression profile when infected with the same species of *Leishmania*. The inherent transcriptional heterogeneity among different THP-1 macrophage cell lines in response to *Leishmania* infection, with consequent variations in functional pathways and gene mechanisms, provides valuable context for interpreting our current results [40]. This raises questions as to when and how *Leishmania* species affect and modify host cell expression. Our results confirm that there is a large number of genes involved in this process, considering that the parasite–host interaction induces changes in gene expression in several models already studied [41]. *L. major* then seems to trigger normal gene expression mechanisms in THP-1 macrophages, i.e., *L. major* seems to induce a gene expression pattern in macrophages equivalent to the uninfected state, thus preventing the development of a strong immune response against the parasite. This suggests an escape mechanism favoring the survival of the parasite and spread even within a cell of the immune system [42].

The lack of differential expression in Predicted Genes at both 12 h and 24 h suggests a potential *Leishmania*-mediated manipulation of host cell gene expression. This might involve maintaining baseline expression levels of crucial immune response genes to establish a favorable environment for parasite survival. The observed decrease in DIAmond Predicted Gene expression at 24 h might indicate additional regulatory mechanisms employed by the parasite.

## 4. Conclusions

This study’s novel methodology yielded results that help comprehend *Leishmania* infection. First, we displayed an exceptional capacity for public data acquisition and processing, employing enrichment analysis and Cytoscape for comprehensive filtering and analysis of leishmaniasis. This approach culminated in identifying and validating six “Predicted Genes” exhibiting relevance to leishmaniasis. Subsequent biological validation revealed that *L. major* maintains basal expression of key immune response-related genes within the utilized model. This observation warrants further investigation into the parasite’s regulatory mechanisms governing the expression of these genes. Additionally, our findings suggest a highly efficacious intracellular escape mechanism employed by *L. major*, contributing to its persistence within host cells. These results allow for exploring the parasite’s immune evasion strategies, and improving the expression of those genes can be one target for disease treatment.

## 5. Materials and Methods

### 5.1. Data Source and Filtering

We selected the following databases: GWAS (Genome-Wide Association Studies), UniProt (Universal Protein Resource), Malacards (*Leishmania*sis Knowledgebase), DisGeNet (Disease-Gene Network), Humanmine (Human Disease-associated Genes and Mutations), and NCBI (National Center for Biotechnology Information). To conduct our searches, the keyword “*Leishmania*sis” was employed, yielding results encompassing microarray and RNAseq data concerning gene expression, DNA methylation, miRNAs, and immune response. The data collected for analysis encompassed genes, proteins, and transcripts associated with the word “leishmaniasis” in general. However, we exclusively focused on data about humans. By adhering to these criteria, we effectively selected results that align with our research objectives, subsequently delving into exploring expressed genes and networks induced by leishmaniasis in human macrophages. Each selected result underwent filtration following the criteria above. Subsequently, the UniProt and HGNC databases were utilized to convert the results into protein and gene symbols.

### 5.2. Enrichment Analysis

This approach assesses whether the gene set is statistically enriched with genes belonging to specific pathways or functional categories, such as those defined by Gene Ontology (GO) terms [43]. To perform the enrichment analysis, we employed the ShinyGO [44] tool, allowing us to interrogate the 287 identified genes and assess their enrichment in Gene Ontology (GO) terms across the three main categories: Biological Process, Molecular Function, and Cellular Component. Within the Biological Process, we focused on terms describing ordered assemblies of molecular functions that contribute to the accomplishment of specific biological functions. We deemed them enriched when the GO term had a false discovery rate (FDR) of 0.05. On enrichment analysis, the FDR was calculated based on the nominal *p*-value derived from the hypergeometric test. Fold enrichment captures the proportion of genes within your list belonging to a given pathway, expressed as a percentage relative to the background set. FDR quantified the probability of observing any given level of enrichment by chance, with larger pathways generally exhibiting lower FDRs due to enhanced statistical power. Fold enrichment, conversely, served as an effect size measure, indicating the degree to which genes in a particular pathway are overrepresented. Pathway enrichment analysis was restricted to a user-defined size range. Following analysis, pathways were first filtered based on a specified FDR threshold and successively selected for the top pathways according to FDRs. Finally, highly redundant pathways sharing over 95% gene overlap were represented solely by the most statistically significant one. The network approach employed by the ShinyGO tool visually depicts the relationships between enriched pathways, with intersecting genes represented by links between pathway nodes. This network visualization allowed the identification of the most biologically relevant processes emerging from the enrichment analysis based on their interconnectedness and shared-gene content. As pathways with greater overlap in gene membership are linked more closely, GO pathways (nodes) are connected if they share 20% or more genes. Darker nodes are more significantly enriched gene sets. Bigger nodes represent larger gene sets. Thicker edges represent more overlapped genes.

### 5.3. Network Analysis

To integrate and assemble PPIs, we used the Cytoscape [17] software (version 3.9.1). We chose two different plug-ins, APID2NET [18] and BioGRID [19], to measure and analyze the PPIs, investigating the genes. The Apid (Agile Protein Interactomes DataServer) reports information on experimental validation, allowing selection and filtering at different quality levels for each protein–protein relationship. This provides access to the interactome of specific species; we focused on human data, more than 90,000 different proteins, and more than 670,000 unique interactions. We also used the BioGRID Cytoscape’s plug-in to investigate protein–protein interaction networks. It currently has more than 70,000 publications, more than 1 million protein and gene interactions, more than 20,000 chemical associations, and more than 800,000 post-transcriptional modifications from diverse species. By using these two plug-ins together, we meant to assemble an interactome with a large quantity of quality data resulting from our HDs. The DIAmonD [21] (Disease Module Detection Algorithm) tool is used to identify “Predicted Genes” within interactome modules. Predicted genes are genes that play a crucial role in the interactome and are highly likely to act as central modulators of the response within the interactome. The DIAmonD algorithm operates by assessing the significance of connectivity among all immediate neighbors of the proteins at each step. The node with the most significant connectivity (i.e., the lowest *p*-value) is integrated into the module, thus expanding the module by one node at each refining step.

In this manner, the Predicted tool facilitates the identification of Predicted Genes that hold potential importance within the assembled PPI network and, consequently, within the host immune response process.

### 5.4. Biological Validation

In this study, we performed biological validation through cellular and molecular methodologies to corroborate the data obtained in silico. We cultured THP1 cells, a human monocyte lineage, as described in the literature [45,46,47]. THP1 cells were cultured in RPMI 1640 medium supplemented with 10% inactivated fetal bovine serum (FBS), 10 mM sodium pyruvate, 10 mM L-glutamine, and gentamicin at 37 °C and 5% CO_2_. To induce macrophage differentiation, THP1 cells were plated in a 24-well plate covered by glass slides and were treated with 10 ng/mL phorbol 12-myristate 13-acetate (PMA) for 48 h.

Successively, the adherent cells were washed to remove excess PMA and infected with metacyclic *L. major* labeled with CFSE in a 10:1 ratio (10 parasites per macrophage) in 24-well plates over glass coverslips. The infection was carried out at 32 °C for 3 h. The cells were then washed to remove non-phagocytized *Leishmania*, and the culture was maintained for 12 h or 24 h. *Leishmania major* (WHO MHOM/IL/80/Friedlin) was grown in Grace’s medium supplemented with 20% FBS, 2 mL glutamine, and gentamicin at 25 °C. Metacyclic promastigote forms were separated by Ficoll gradient on day 5 of culture. In a 15 mL tube, 2 mL of 20% Ficoll was added, followed by 2 mL of 10% Ficoll. The *Leishmania* suspension was then added carefully to the wall of the tube, forming a third phase. The triphasic mixture was centrifuged at 800 g for 10 min at 4 °C. The ring formed, and all the supernatant above it, corresponding to the 10% Ficoll phases, was collected as it contained the metacyclic forms. The *Leishmania* was then washed three times, resuspended in complete RPMI, and incubated with CFSE as first described. For the infection index analysis, the coverslips were removed, washed, and colored with Wright–Giemsa staining (Thermo Fisher, Waltham, MA, USA), and the image was acquired using an optical microscope at 12 and 24 h post-infection (hpi). For fluorescent images, the coverslips were incubated in PBS pH 7.2/0.02% of DAPI. The cells were examined under a Zeiss Imager Z2 fluorescent microscope under a 20× lens. The cells were lysed with Trizol, and RNA was extracted following the manufacturer’s protocol (Invitrogen, Carlsbad, CA, USA). RNA quality was assessed using the NanoDrop One equipment (Thermo Fisher Scientific, WI, USA) and evaluating the 260/280 and 260/230 ratios. To evaluate the expression of the genes of interest, we performed quantitative reverse transcription PCR (RT-qPCR). We performed this technique in three steps: (i) conversion of RNA into complementary DNA (cDNA) through reverse transcription, (ii) amplification of cDNA using the polymerase chain reaction (PCR), and (iii) quantification of the amplification of gene expression products in real-time [48].

To eliminate DNA contamination in RT-qPCR experiments, we maintained separate areas, dedicated equipment, and high-quality reagents for each step. Sterile techniques were employed when handling samples and reagents. Additionally, we included a negative control (no-template control) and a no-RT control (without reverse transcriptase).

Standard procedures were followed in our laboratory to prevent DNA contamination. We implemented specific measures to ensure the cleanliness of the materials used, including the use of separate pipettes and filtered pipette tips exclusively for RT-qPCR. All surfaces involved in RT-qPCR reactions were decontaminated with a 5% bleach solution, which was left for 10–15 min before being wiped with de-ionized water. In every RT-qPCR experiment, a crucial no-RT (–RT) control was included. This control is essential for detecting potential genomic DNA contamination, thereby ensuring the accuracy of our results by helping differentiate RNA-specific signals from any genomic DNA background. We also employed a specific procedure to remove DNA after RNA extraction, applying DNase I (Thermo Fisher) to eliminate genomic DNA contamination prior to reverse transcription. The manufacturer’s protocol was followed for DNase treatment, using 1 µL/µg and incubating at 37 °C for 30 min. For statistical analysis of RT-qPCR results, we used the Comparative delta–delta CT Method for Relative Quantification (ddCT). This method uses the arithmetic formula 2-CT to achieve the result for relative quantification. The amplification efficiency of the target of interest and the amplification efficiency of the reference (endogenous control) must be approximately equal for the comparative CT method to be validated. To perform RT-qPCR, we employed specific primers for the Predicted Genes (Supplementary Table S3), which were designed based on information from the PrimerBank database [49]. We also used the control (endogenous) expression gene, GAPDH. The Applied Biosystems 7900HT System equipment, along with Applied Biosystems SDSv2.4 software, was used for data visualization and preliminary analysis of the obtained results. The cellular lysis process involved TRIzol Reagent (Invitrogen) following the manufacturer’s protocol. After lysing THP-1 cells infected with *Leishmania major*, RNA isolation was performed using chloroform to separate RNA from phenol. Isopropanol was then used to isolate RNA, resuspended in RNAse-free water. The quality of the RNA was assessed using NanoDrop before proceeding with cDNA synthesis. For cDNA synthesis, the RevertAid First Strand cDNA Synthesis Kit (Thermo Fisher) was used to generate the necessary cDNA for RT-qPCR. To form the RT-qPCR mix, we used the PowerUp SYBR Green Master Mix (Thermo Fisher) and the primers (listed in Table S4), following the manufacturer’s protocol. This mix, along with the samples, was plated on the MicroAmp Optical 96-Well Reaction Plate (Applied Biosystems, Foster City, CA, USA) and sealed using the MicroAmp Optical Adhesive Film (Applied Biosystems). The subsequent RT-qPCR was conducted with the 7900HT System (Applied Biosystems).

## Data Availability

Data generated in this study are available from the corresponding author upon request.

## Supplementary Materials

The following supporting information can be downloaded at: https://www.mdpi.com/article/10.3390/ijms252212084/s1.
