# Supplementary material for: Expression of Network Medicine-Predicted Genes in Human Macrophages Infected with Leishmania major
Source: Int J Mol Sci. 2024 Nov 11;25(22):12084. doi: 10.3390/ijms252212084 (PMC11594204; doi:10.3390/ijms252212084)

Supplementary Material

Table S1

Summary of Datasets				
**Human Reference Dataset**	**Original Database**	**Reference**	**Source Data**	**Number of retrived proteins**
**HD1**	GWAS	EBI GWAS Catalog (www.ebi.ac.uk/gwas/)	Database	14
**HD2**	UNIPROT	Uniprot (www.uniprot.org)	Database	4
**HD3**	Malacards DB - Leishmaniasis	MalaCards-the human disease database (http://www.malacards.org)	Database	17
**HD4**	Malacards DB - Cutaneous Leishmaniasis	MalaCards-the human disease database (http://www.malacards.org)	Database	9
**HD5**	Malacards DB - Mucocutaneous Leishmaniasis	MalaCards-the human disease database (http://www.malacards.org)	Database	5
**HD6**	Malacards DB - Visceral Leishmaniasis	MalaCards-the human disease database (http://www.malacards.org)	Database	8
**HD7**	Malacards DB - Cutaneous Leishmaniasis - Downreg. Genes	MalaCards-the human disease database (http://www.malacards.org)	Database	20
**HD8**	Malacards DB - Cutaneous Leishmaniasis - Upreg. Genes	MalaCards-the human disease database (http://www.malacards.org)	Database	51
**HD9**	Disgenet - Leishmania	Disgenet-database of gene-disease association (http://www.disgenet.org)	Database	46
**HD10**	Disgenet - Cutaneous Leishmania	Disgenet-database of gene-disease association (http://www.disgenet.org)	Database	45
**HD11**	Manual literature curation	REFERENCES	Literature	57
**HD12**	Humanmine - KEGG Leishmaniasis pathway	Humanmin-integrated database of Homo sapiens genomic data (http://www.humanmine.org)	Database	73
**HD13**	NCBI - OMIM	NCBI OMIM database (www.ncbi.nlm.nih.gov/omim)	Database	5
**HD14**	NCBI - Nucleotide	NCBI Nucleotide database (www.ncbi.nlm.nih.gov/nuccore/)	Database	27
**HD15**	NCBI - Gene	NCBI Gene database (www.ncbi.nlm.nih.gov/gene)	Database	51
**HD16**	NCBI - Protein	NCBI Protein database	Database	7

Table S2

Genes					
ABCA8	CXCL5	HLA-DOB	IRAK4	NFKBIB	SPTA1
ACO1	CXCL8	HLA-DPA1	IREB2	NKG7	SPTB
ADAMDEC1	CXCL9	HLA-DPB1	ITGA4	NLRP3	SST
AKT1	CXCR1	HLA-DQA1	ITGAM	NOS2	STAT1
AQP1	CXCR2	HLA-DQA2	ITGAX	NR0B2	STAT3
AQP8	CXCR3	HLA-DQB1	ITGB1	NR1I2	STAT6
ARG1	CYBA	HLA-DRA	ITGB2	OGN	TAB1
ATOH7	CYBB	HLA-DRB1	JAK1	OR2T4	TAB2
BCAR1	DCAF7	HLA-DRB3	JAK2	OR6A2	TDO2
BCL2	DEFA6	HLA-DRB4	JCHAIN	P2RX7	TFRC
BEST4	DLL1	HLA-DRB5	JUN	PGD	TGFB1
BPIFC	DPP4	HLA-E	KIR2DL3	PIK3CA	TGFB2
C1QB	ELK1	HSPA1L	KLRK1	PIK3CG	TGFB3
C3	ENTPD3	HSPA4	LAG3	PITRM1	TLR2
CA7	ENTPD8	IDO1	LAMP1	PKD2L1	TLR3
CARD11	EPSTI1	IER3	LAMP2	PPARG	TLR4
CCL19	EXOSC7	IFNAR1	LAMTOR3	PRDX2	TLR7
CCL2	F2R	IFNAR2	LCN2	PRF1	TLR8
CCL20	FAM20C	IFNG	LMLN	PRKCB	TLR9
CCL24	FAS	IFNGR1	LTA	PSMB6	TMCO5B
CCL3	FASLG	IFNGR2	LYZ	PTGS2	TNF
CCL3L1	FASN	IGF1	MAP3K7	PTPN1	TNFRSF18
CCL3L3	FCER1G	IGLL1	MAPK1	PTPN2	TNFRSF4
CCL4	FCER2	IKBKB	MAPK11	PTPN6	TRAF1
CCL4L1	FCGR1A	IL10	MAPK12	PYY	TRAF2
CCL4L2	FCGR1B	IL10RA	MAPK13	RAB5A	TRAF3
CCL5	FCGR2A	IL12A	MAPK14	RAB7A	TRAF5
CCL7	FCGR2C	IL12B	MAPK3	RARRES3	TRAF6
CCL8	FCGR3A	IL13	MAPK8	REG1A	TRIM63
CCR5	FCGR3B	IL15	MAPK9	REG1B	TTLL5
CCR7	FCN2	IL17A	MARCKSL1	REG3A	UBD
CD209	FH	IL18	MBL2	REL	UCN2
CD3D	FLI1	IL18BP	MIF	RELA	VAMP3
CD40	FLII	IL1A	MMP1	RORC	VAMP8
CD40LG	FOS	IL1B	MMP2	ROS1	VSTM2A
CD8A	FOXP3	IL1RL1	MMP3	S100A8	WARS
CHUK	G6PD	IL2	MON2	S100A9	YY1
CLEC4D	GBP1	IL21	MPI	SCGB2A2	
COL1A1	GBP4	IL22	MPL	SEC22B	
CR1	GBP5	IL27	MROH9	SELL	
CREBBP	GNLY	IL2RA	MT-CYB	SERPINA1	
CRP	GTF3C1	IL2RB	MT1M	SLC11A1	
CSNK1A1	GZMA	IL3	MTOR	SLC52A2	
CTLA4	GZMB	IL32	MYD88	SLC6A4	
CTSB	GZMH	IL33	NCF1	SLPI	
CXCL1	GZMK	IL4	NCF2	SNX10	
CXCL10	HLA-A	IL5	NCF4	SOCS1	
CXCL11	HLA-B	IL6	NEUROD1	SOCS3	
CXCL2	HLA-DMB	INSL5	NFKB1	SOX5	
CXCL3	HLA-DOA	IRAK1	NFKBIA	SPIB	

Table S3

SHARED APID -Biogrid									
ABL1	CBLB	ESR1	IKBKE	MAP3K1	PAG1	NFKB2	RASA1	SYK	VCP
ABL2	CD247	FADD	IKBKG	MAP3K11	PAK1	NR3C1	RET	TANK	WAS
ADAM15	CD38	FGFR1	IL4R	MAP3K14	MAP3K1	PAG1	RIPK1	TBK1	XIAP
APPL1	CD3E	FGR	IL6ST	MAP3K2	MAP3K11	PAK1	RIPK2	TEC	YES1
AR	CDC25C	FOXO3	INPP5D	MAP3K3	MAP3K14	PDCD6IP	RIPK3	TGFBR1	YWHAB
ARHGAP32	CDC37	FRS2	INPPL1	MAP3K5	MAP3K2	PDGFRA	RPS6KA3	TGFBR2	YWHAE
ARRB1	CDKN1B	FYB	INSR	MAP3K8	MAP3K3	PDGFRB	SH2B2	TICAM1	YWHAG
ARRB2	CFLAR	FYN	IRS1	MAP4K1	MAP3K5	PECAM1	SH2D1A	TIRAP	YWHAQ
ASAP1	CRK	GAB1	IRS2	MAPK8IP1	MAP3K8	PIK3CB	SH2D2A	TNFAIP3	YWHAZ
AXL	CRKL	GAB2	IRS4	MAPK8IP3	MAP4K1	PIK3R1	SH3BP2	TNFRSF10A	ZAP70
BCL10	CSF1R	GAB3	ITCH	MAPT	MAPK8IP1	PIK3R2	SH3KBP1	TNFRSF10B	
BCL3	CSF2RB	GHR	ITK	MAVS	MAPK8IP3	PIK3R3	SHB	TNFRSF11A	
BCR	CSK	GRAP2	JAK3	MBP	MAPT	PLCG1	SHC1	TNFRSF1A	
BID	CTNNB1	GRB10	KHDRBS1	MDM2	MAVS	PLCG2	SKAP2	TNIP2	
BIRC2	CYLD	GRB2	KIT	MDM4	MBP	PRKCA	SLA	TNK2	
BIRC3	DNAJA3	GSK3B	LAT	MET	MDM2	PRKCD	SMAD3	TP53	
BLNK	DOK1	HCK	LCK	MST1R	MDM4	PRKCQ	SMAD7	TRADD	
BMX	DOK2	HDAC3	LCP2	MUC1	MET	PRKCZ	SNCA	TRAF3IP2	
BRAF	DUSP1	HDAC6	LRRK1	NCK1	MST1R	PTEN	SOS1	TUBA1A	
BTK	EGFR	HDAC7	LRRK2	NCK2	MUC1	PTK2	SOS2	TUBB	
BTRC	EP300	HIF1A	LYN	NCOA1	NCK1	PTK2B	SQSTM1	TYK2	
CASP10	EPOR	HSP90AA1	MALT1	NCOA3	NCK2	PTPN11	SRC	UBASH3B	
CASP3	ERBB2	HSP90AB1	MAP2K1	NEDD4	NCOA1	PTPN22	STAP2	UBC	
CASP8	ERBB3	HSPA1A	MAP2K4	NEDD9	NCOA3	PXN	STAT5A	UCHL1	
CAV1	ERBB4	HSPA8	MAP2K6	NFKB2	NEDD4	RAF1	STAT5B	VAV1	
CBL	ERRFI1	IGF1R	MAP2K7	NR3C1	NEDD9	RAPGEF1	STUB1	VAV3	

Table S4

Selected genes	
BIRC2	NCK2
CASP8	NFKB2
CAV1	RIPKC2
EPOR	RPS6KA3
FOXO3	SMAD7
GAB1	TANK
IL4R	TGFBR1
IL6ST	TNFAIP3
INSR	TNFRSF10A
LCP2	WAS
K4	XIAP

Table S5

Gene	Forward	Reverse
TNF	CTGCACTTTGGAGTGATCGG	TGAGGGTTTGCTACAACATGGG
IL1B	CACX3ATGCACCTGTACGATCA	GTTGCTCCATATCCTGTCCCT
IL1A	AGATGCCTGAGATACCCAAAACC	CCAAGCACACCCAGTAGTCT
IL6	AGAGGCACTGGCAGAAAAC	TGCAGGAACTGGATCAGGAC
BIRC2	AACTCTTGGCCTTTCATTCG	TGCTTTTGTTGTGATGGTGG

CASP8	AGGCCAGATCTTCACTGTCC	GGTCACTTGAACCTTGGGAA
CAV1	TCCCTTCTGGTTCTGCAATC	CGAGAAGCAAGTGTACGACG
EPOR	CAGGCCAGATCTTCTGCTTC	GACGCTCTCCCTCATCCTC
FOXO3	AGTTCCCTCATTCTGGACCC	CTTCAAGGATAAGGGCGACA
GAB1	TGCGTAACACGAACCATCTC	GTGGTGAAGTGGTCTGCTCC
IL4R	TGATTTCTTCCAGCTGTGTGT	CAGGGGTCCCCCACTTC
IL6ST	CAGCATGAATCCAGTCCAGA	CCCTCAGTACCTGGACCAAA
INSR	GTCGGGCTGAGAACAGTTG	GTCGAACGATGTTGGACTCA
LCP2	GTTTGCGTGTGAAGATGCTC	GACATCCAGAAGTTCCCCAA
MAP3K14	TTCTGGGTGAGGTCAATCCT	GGATGGAGGACAAGCAGACT
NCK2	CTTGATGTCCAGCTCCTGGT	TCACCTCTCAACTGTGTGCC
NFKB2	GGACTGGTAGGGGCTGTAGG	CACATGGGTGGAGGCTCT
RIPK2	CTCTTCAAATGTTCTCAAAACTGG	AGAAAGTGGATGGGCACAAA
RPS6KA3	TGGGTTAATCTCCTCCTCTCC	GTGGCAGAAGATGGCTGTG
SMAD7	CCAGGCTCCAGAAGAAGTTG	CCAACTGCAGACTGTCCAGA
TANK	TTTATTGAGTTGCTCGCCAA	CAAAGGAAGACTTGTAACCTGGA
TGFBR1	TTGTCTTTTGTACAGAGGTGGC	GCTGCTCCTCCTCGTGCT
TNFAJP3	ACAGCTTTCCGCATATTGCT	TTGACCAGGACTTGGGACTT
TNFRSF10A	GGCTATGTTCCCATTGCTGT	AGACCTTCAAGTTTGTCGTCG
WAS	AGCCACTCAGTCATCCCATT	ATGCACGTGATGCAGAAGAG
XIAP	CCTTCAAAACTGTTAAAAGTCATCTTC	GGACCCTCCCCTTGGAC
18srRNA	TCGGAACTGAGGCCATGATT	CTTTCGCTCTGGTCCGTCTT
GAPDH	ACCCACTCCTCCACCTTTGAC	TGTTGCTGTAGCCAAATTCGTT

Figure S1

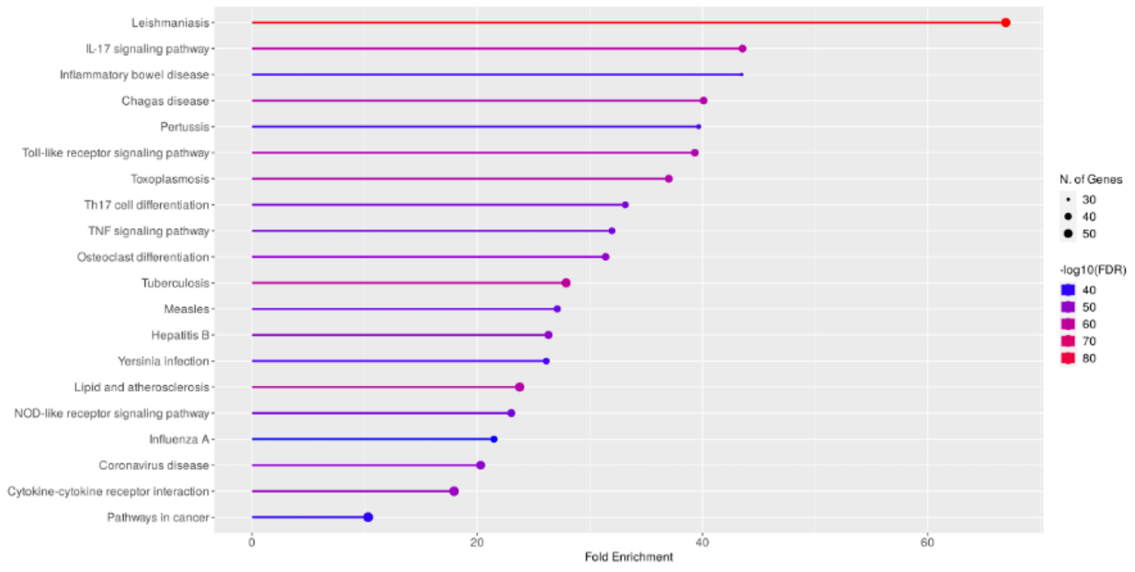

Supplement: Supplementary file 1 [file ijms-25-12084-s001.zip › ijms-3220303-supplementary.pdf]
